# Lee Silverman Voice Treatment to Improve Speech in Parkinson's Disease: A Systemic Review and Meta-Analysis

**DOI:** 10.1155/2021/3366870

**Published:** 2021-12-27

**Authors:** Tingting Pu, Min Huang, Xiangyu Kong, Meng Wang, Xiangling Chen, Xixi Feng, Changyou Wei, Xiechuan Weng, Fan Xu

**Affiliations:** ^1^School of Pharmacy, Dali University, Yunnan 671000, Dali, China; ^2^Department of Public Health, Chengdu Medical College, Sichuan 610500, Chengdu, China; ^3^Department of Physiology, Chengdu Medical College, Sichuan 610500, Chengdu, China; ^4^Beijing Institute of Basic Medical Sciences, Beijing 100850, China

## Abstract

**Background:**

Speech changes occur in the early stages of Parkinson's disease (PD) and cause communication difficulties, leading to social isolation. Lee Silverman voice treatment (LSVT) is a speech therapy approach designed to improve patients' language and voice capabilities.

**Objective:**

The effectiveness of the LSVT was compared with that of other speech interventions or no treatment to evaluate PD patients with dysarthria.

**Design:**

Systematic review with meta-analysis of randomized trials. *Data Sources*: PubMed, Embase, Cochrane Library, CNKI, and SinoMed library were searched from inception to December 2021 related to PD and LSVT.

**Method:**

Abstracts were screened and reviewed against the eligibility criteria (intervention group participants were PD assessed based on LSVT (LSVT Loud) and randomized control).

**Result:**

Ten randomized controlled trials were identified on speech symptoms in patients with PD. Compared with the respiratory therapy (RET) exercise, or no training group, a significant improvement was detected in the sound press level (SPL) after immediate treatment during the reading of vowel and rainbow passages and an increase in semitone standard deviation (STSD). Furthermore, the LSVT training significantly increased the participants' scores on unified Parkinson's disease rating scale (UPDRS-III) and speech intelligibility.

**Conclusion:**

This meta-analysis demonstrated the efficacy of LSVT in increasing vocal loudness and functional communication among individuals with PD. However, most studies included participants with mild-moderate PD. Thus, additional randomized controlled trials (RCTs) with large sample sizes are needed to validate the efficacy of LSVT in patients with different progressions of PD, including severe PD.

## 1. Introduction 

Parkinson's disease (PD) is characterized by the loss of dopamine neurons, leading to motor and nonmotor dysfunction [1]. PD patients reached 5 million in the USA, affecting 1% of above 60-year-old population [2]. PD patients manifest motor symptoms, such as tremor, muscular rigidity, and bradykinesia, while speech disorders are one of the common nonmotor symptoms that make PD patients often experience a reduction in loudness, imprecise articulation, abnormal nasal resonance, voice and pitch, and prosody error symptoms [3]. Phonation is the essential interaction between humans and environments that undertakes human thought and mood [4]. In our previous studies, we have demonstrated that PD patients have head tremble, facial expressions, and speech disorders [5–7]. PD patients with speech disorders have difficulties in speaking or signaling their thoughts and intentions, which causes impairments in social interaction and communication accompanied by psychotic disorders.

Clinically, speech disorders are defined as hypophonia caused by respiration, vocal production, and articulation [8]. PD can be treated with surgical treatments, such as deep brain stimulation of the subthalamic nucleus (STN-DBS) proven to be an effective treatment for limb motor symptoms. However, the data suggest that bilateral STN-DBS (with or without medication) most often deteriorates speech functions that do not improve once the stimulation is turned off [9]. Studies have found that STN-DBS patients treated with Lee Silverman voice treatment (LSVT) had significant clinical improvement in VHI scores, voice, and speech [10]. In addition, the traditional drug treatment was administered, 1 or 2 drugs a day for a month, emphasizing to improve voice clarity and prosody to determine the correct position of phonemic phonation and produce language-specific phonemics [11, 12]. It also increases the intermuscular coordination and intensity exercise of the tongue, chin, mouth, and other organs but has only modest effects on the prosodic aspects of parkinsonian speech [13]. Jan Rusz has expressed similar views; the long-term administration of dopamine in PD patients can stabilize the severity of speech disorder and improve speech performance [14].

## 2. Methods

### 2.1. Search Strategy

Two reviewers (Tingting Pu and Xinagyu Kong) independently searched the PubMed, Embase, Cochrane Library, CNKI, and SinoMed library databases up to December 2021, using various speech disorder-related words and MeSH terms in combination with PD, irrespective of date, language, region, or publication type. MeSH search terms included PD. Free words included voice/speech therapy, voice/speech treatment, voice/speech training, voice/speech rehabilitation, or LSVT. The search was limited to published clinical studies.

### 2.2. Inclusion and Exclusion Criteria

**Figure 1:**
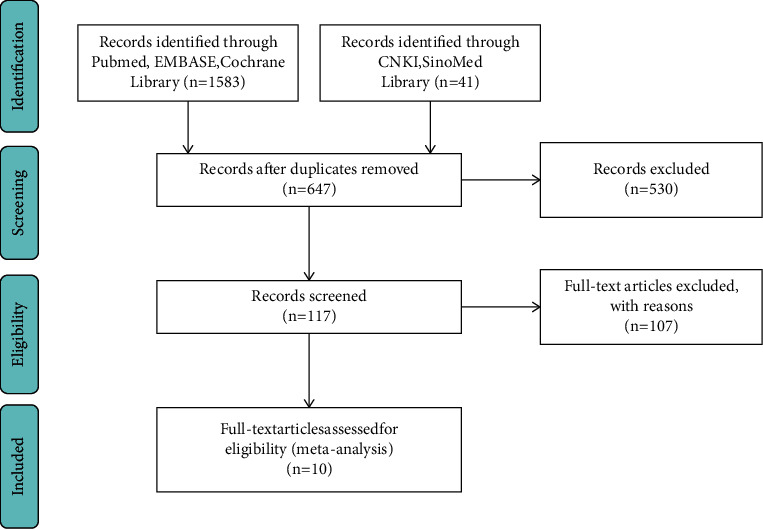
Flowchart displaying study selection.

Inclusion criteria were as follows: (1) All trial types are limited to randomized controlled trials (RCTs); (2) patients' age, sex, drug type, duration of illness, duration of treatment, and voice handicap index (VHI) were not limited, but only included the PD patients who met the UK Parkinson's disease society bank criteria; (3) intervention: LSVT; (4) control group: using other speech disorder treatments or no intervention measures; and (5) main outcomes include sound press level (SPL) and VHI, and the secondary outcomes consist of the semitone standard deviation (STSD), unified Parkinson's disease rating scale-III (UPDRS-III)–speech item score and speech intelligibility. The exclusion criteria were no outcomes described, no control groups, or animal experiments (Figure 1).

### 2.3. Data Extraction

Two authors (Tingting Pu and Xiangyu Kong) independently extracted the demographic data and treatment information, and the disagreements were resolved by a third author (Min Huang). The baseline information was extracted from 10 studies: the first author's name, year of publication, title, design type, study subjects (number, age, male/female ratio), disease degree, and length of the disease (Table 1). In addition, the intensity, course of treatment, and follow-up time were extracted from the intervention measures. The primary outcomes included the SPL and VHI, and the secondary outcomes consisted of the STSD, UPDRS-III, speech item score, and speech intelligibility.

### 2.4. Quality Assessment

The type of trial, allocation concealment, blinding of subjects, blinding of results, loss of follow-up bias, selection bias, and other biases were involved in assessing the methodological quality. The risk of bias was assessed using the Cochrane risk-of-bias assessment tool.

### 2.5. Statistical Analysis

The meta-analysis was performed with the statistical software review manager (version 5.4, UK). We defined the mean, standard deviation (SD), and OR with 95% confidence interval (CI) as the effect size. Heterogeneity was assessed by Cochrane's Q statistics (chi-square) or inverse variance (*I*^2^). If *I*^2^ was <50%, and the *P*-value was >0.1, these studies could be considered homogeneous as assessed by a fixed-effects model; or else *I*^2^ ≥ 50%, *P* < 0.10, the random effect model was used for meta-analysis. When heterogeneity was high (*I*^2^ ≥ 50%, *P* < 0.10), subgroup analysis was applied to analyze the sensitivity.

## 3. Results

### 3.1. Study Selection and Characteristics

**Figure 2:**
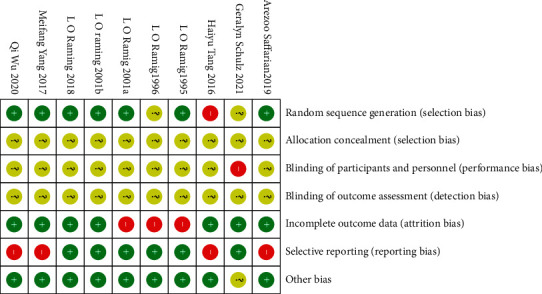
Risk-of-bias summary: a review of authors' judgments about each risk-of-bias item for each included study.

**Figure 3:**
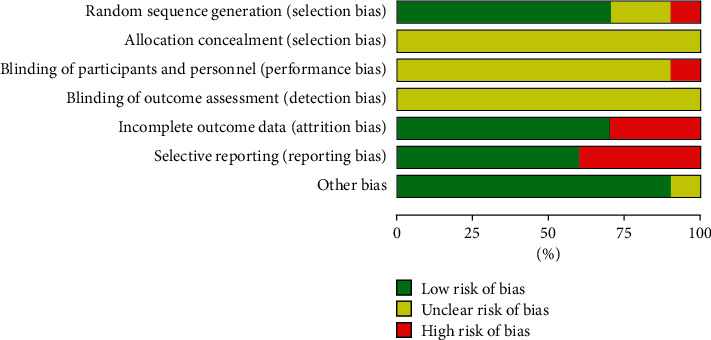
Risk-of-bias graph: a review of authors' judgments about each risk-of-bias item presented as percentages across all included studies.

A total of 10 articles were ruled out from 1624 references. The detailed information for literature screening was as follows (Figure 1). Table 1 summarizes the demographic data of 269 patients in the intervention group and 240 patients in the control group. Descriptive statistics were computed to identify the demographic information, including intervention, Hoehn–Yahr (HY) score, PD duration, age, sex, and outcomes (Table 1). In addition, the risk-of-bias was assessed using the Cochrane handbook based on published research and registration trials (Figures 2 and 3).

### 3.2. SPL Immediately after Treatment

**Figure 4:**
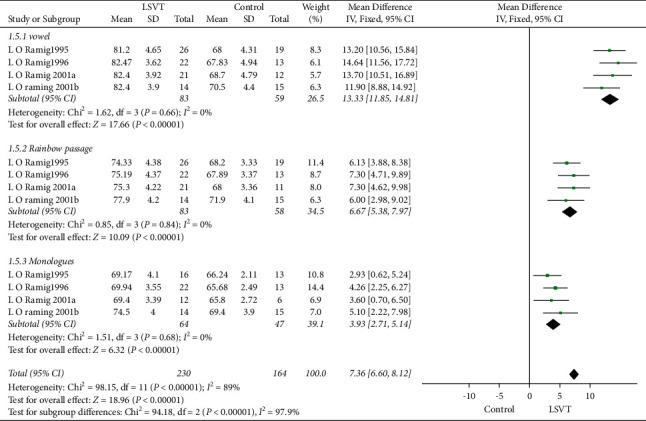
SPL immediately after the treatment during a reading of vowel, rainbow passage, and monologues.

We conducted four types of studies (LO Ramig 1995, LO Ramig 1996, LO Ramig 2001a, LO Ramig 2001b) to evaluate the efficacy of SPL. A total of 141 participants were tested with three voice tasks, and voice testing revealed a higher SPL level in the LSVT group (7.36 dB, 95% CI: 6.60–8.12, *P* < 0.00001) than the control group, with high heterogeneity (*I*^2^ = 89%). After subgroup analyses, the SPL level increased during pronunciation vowel (13.33 dB, 95% CI: 11.85–14.81, *P* < 0.00001), while reading of the rainbow passage (6.67 dB, 95% CI: 5.38–7.97, *P* < 0.00001), the monologues (3.93 dB, 95% CI: 2.71–5.14, *P* < 0.00001), and the heterogeneity was not significant across four studies (from 89% to 0%) (Figure 4).

### 3.3. SPL for the Different times after Treatment

**Figure 5:**
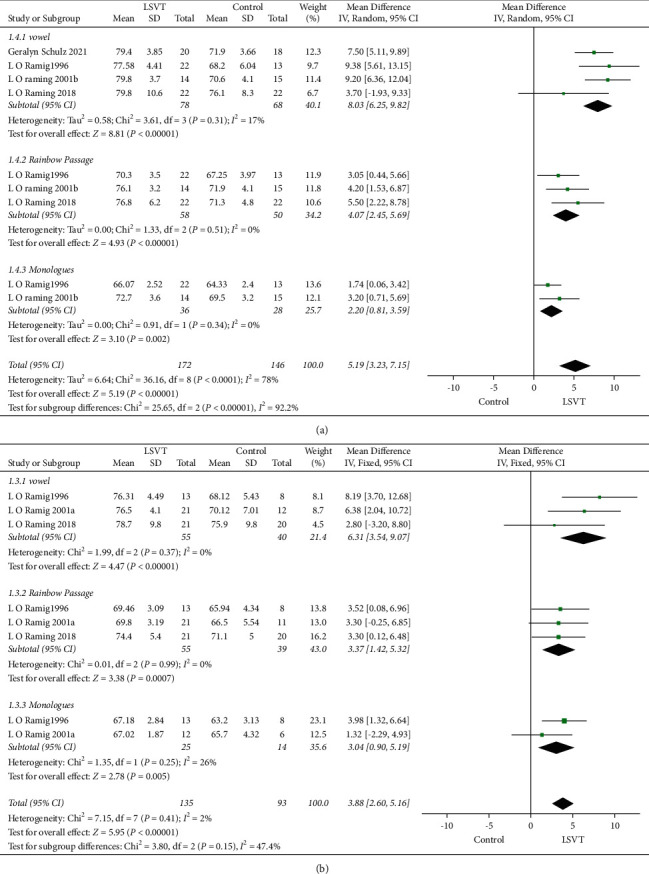
Forest plot showing mean difference and 95% CI of SPL at different assessment times. (a) 1–6 months. (b) 6–12 months.

According to the different SPLs after treatment in the studies, the test time was divided into 1–6 months and 6–12 months. Compared with the control group, the LSVT group had an improved SPL score 5.19 dB (95% CI: 3.23–7.15, *P* < 0.00001) after 1–6 months (Figure 5(a)) and 3.88 dB (95% CI: 2.60–5.16, *P* < 0.00001) after 6–12 months (Figure 5(b)). Four studies (L O Ramig 2001b, L O Ramig 2018, L O Ramig 1996, Geralyn Schulz 2021) reported that the higher SPL scores during pronunciation vowel, reading of the rainbow passage, and monologues were 8.03 dB (95% CI: 6.25–9.82, *P* < 0.00001), 4.07 dB (95% CI: 2.45–5.69, *P* < 0.00001), and 2.20 dB (95% CI: 0.81–3.59, *P*=0.002), respectively, after 1–6 months. In addition, three studies (L O Ramig 2001a, L O Ramig 2018, LO Ramig 1996) reported an increased SPL score during pronunciation vowel, reading of the rainbow passage, and monologues 6.31 dB (95% CI: 3.54–9.07, *P* < 0.00001), 3.37 dB (95% CI: 1.42–5.32, *P*=0.0001), and 3.04 dB (95% CI: 0.90–5.19, *P*=0.005), respectively, after 6–12 months (Figure 5(b)).

### 3.4. VHI Effect after Treatment

**Figure 6:**
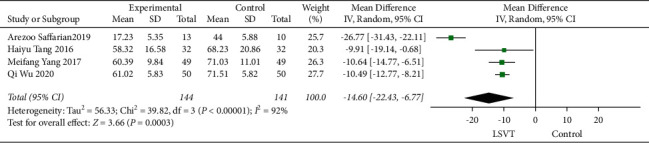
VHI after immediate treatment.

Four studies, Arezzo Saffarian 2019, Qi Wu 2020, Haiyu Tnag 2016, and Meifang Yang 2017, were included. Importantly, we found a decreased grade of VHI by the LSVT treatment (−14.60, 95% CI: −22.43 to −6.77, *P* < 0.00001) compared to the control (Figure 6).

### 3.5. STSD during a Reading of the Rainbow and Monologues

**Figure 7:**
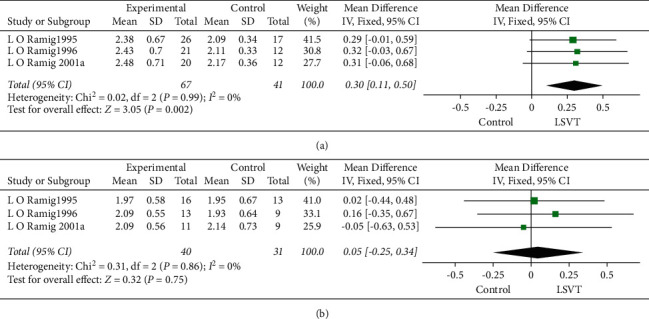
Forest plot showing mean difference and 95% CI of STSD during rainbow passage (a) and monologues (b).

The STSD was described only in three studies (LO Ramig 1995, LO Ramig 1996, LO Ramig 2001a). Interestingly, we compared the score of STSD in patients with PD and controls and found that the LSVT improved the score during reading the rainbow passage (0.30 dB, 95% CI: 0.11–0.50, *P*=0.002) (Figure 7(a)). However, no significant difference was detected during the monologues (*P*=0.75) (Figure 7(b)).

### 3.6. UPDRS-III Speech Item Score

**Figure 8:**
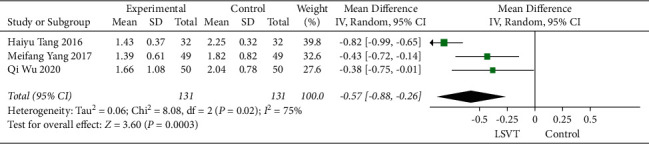
Forest plot showing mean difference and 95% CI of UPDRS-III speech item score after immediate treatment.

Patients with PD included in Qi Wu 2020, Haiyu Tang 2016, and Meifang Yang 2017 were subjected to a voice test. Compared to the control, the UPDRS-III speech item score was significantly reduced after the treatment (−0.57, 95% CI: −0.88 to −0.26, *P*=0.0003) with significant heterogeneity (*I*^2^ = 75%) (Figure 8).

### 3.7. Speech Intelligibility Effect after Treatment

**Figure 9:**
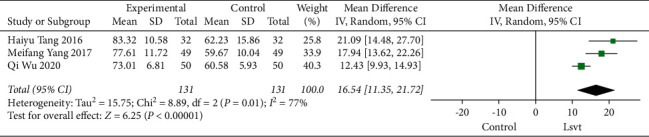
Forest plot showing mean difference and 95% CI of speech intelligibility immediately treatment.

In this study, three studies (Haiyu Tang 2016, Meifang Yang 2017, and Qi Wu 2020) of data were used to analyze speech intelligibility in patients. The current data indicated that LSVT improves the speech intelligibility of PD (16.54, 95% CI: 11.35–21.72, *P* < 0.00001). However, the meta-analysis revealed significant heterogeneity (*I*^2^ = 77%) (Figure 9).

### 3.8. Sensitivity Analysis

Considerable heterogeneity (*I*^2^ = 75%) was observed after immediate treatment in the UPDRS-III speech item score. The leave-one-out sensitivity analysis revealed a decrease (from 75% to 0%) in heterogeneity and UPDRS-III speech item score (−0.41, 95% CI: −0.64 to −0.19, *P*=0.0004) when one study (Tang 2016) was excluded. Compared to the baseline data, interventions, and the evaluation of outcomes in Tang 2016, those in other studies in this meta-analysis were not considerably different. Therefore, the heterogeneity may be derived from systematic errors. In addition, heterogeneity (*I*^2^ = 92%) in the VHI was attributed to another study (Saffarian 2019). When this study was excluded, a decrease (from 92% to 0%) and a reduction of the VHI score (−10.50, 95% CI: −12.45 to −8.45, *P* < 0.00001) indicated that the heterogeneity was attributable to this study (Saffarian 2019).

## 4. Discussion

Speech changes occur in the early stages of PD and cause communication difficulties, leading to social isolation [15]. Typically, pharmacological and neurosurgical treatments are not effective on the prosodic aspects of parkinsonian speech [16–18]. Over the years, speech and language pathologists presented various methods such as the SLTs to improve communication in PD patients [19]. The LSVT is a speech and language therapy method and one of the most widely used speech intervention methods in hypokinetic dysarthria associated with PD [20], wherein the patients are asked to produce a loud voice and focus their efforts on attaining, monitoring, and maintaining the loud voice. The objective of this meta-analysis was to evaluate the effectiveness of LSVT compared to other speech interventions or no treatment for PD patients with dysarthria.

During the past years, several studies investigated the effectiveness of LSVT on PD patients with dysarthria. To evaluate the effects of LSVT on dysphonia in patients with PD, we analyzed the data from published RCTs. After strict screening, ten published articles were included in this study [21–30]. The meta-analysis of SPL from six trials (*n* = 211) and the VHI from four tests (*n* = 288) found LSVT to be more effective than other speech interventions or no treatment to improve vocal loudness and voice handicap. In addition, the follow-up results of SPL indicated long-term effects of LSVT and the LSVT improved UPDRS-III speech item score and speech intelligibility among the PD patients with dysphasia problems. In 2020, a consequence showed that compared with LSVT ARTICT, PD patients treated with LSVT showed more significant increases from baseline to posttreatment in transcription [31]. Furthermore, research has demonstrated that STN-DBS's impact on speech is variable and multifactorial, with most patients exhibiting a decline of speech intelligibility [32]. Jennifer Spielman further found that, compared with only LSVT or STN-DBS, several treated individuals with combination therapy had better significant clinical improvement in VHI scores and more variable long-term maintenance [10]. These findings proved that the LSVT had good responses and long-term effects than either other speech intervention, surgical operational, or no intervention. In addition, the long-term administration of dopamine in PD patients with the phonatory-prosodic subtype can stabilize the severity of speech disorder and improve speech performance [14]. The combined treatment of LSVT and levodopa remains to be explored.

A previous study by Yuan et al. evaluated the effectiveness of LSVT. There were some differences between the previous and current studies. The current meta-analysis included more studies compared to the study by Yuan et al. Moreover, some factors, such as the publication bias that might influence the meta-analysis results, were assessed in our study. In addition, the outcome indicators of the current study included SPL, VHI, STSD, and UPDRS-III speech item scores, but those in the study by Yuan et al. only included SPL and VHI [33]. Despite these differences, we also found that the LSVT had good responses compared to either speech interventions or no intervention and had long-term effects, consistent with Yuan et al. study, suggesting the effectiveness of LSVT.

Nevertheless, the present meta-analysis has some limitations. First, we used stringent criteria for study inclusion and then performed data extraction and analysis. Heterogeneity was a significant issue while interpreting the results of the present meta-analysis. In the overall analysis of VHI after the treatment, we found high heterogeneity between the studies. We found high heterogeneity between the reflections in the comprehensive analysis of the UPDRS-III speech item score after the treatment. After leave-one-out sensitivity analyses, heterogeneity was removed after the exclusion of the study (Tang 2016). After investigation, the heterogeneity may be derived from systematic errors. Second, the participants in all studies were volunteers or recruited from outpatient clinics, support groups, and physicians motivated to improve their motor performance and were not the accurate representation of the broad population with PD. Third, although the included studies were highly consistent in methodology, differences may exist in relative effects on the meta-analysis results. To assess the impact of varying stages of disease progression on the effects of LSVT on dysarthria, we followed the Cochrane Handbook for Systematic Reviews of Interventions to ensure the accuracy of the meta-analysis results. Nevertheless, we followed the Cochrane Handbook for Systematic Reviews of Interventions to ensure the accuracy of the meta-analysis results.

## 5. Conclusion

This study demonstrated the efficacy of LSVT in increasing vocal loudness and functional communication among individuals with PD. However, most studies included participants with mild-moderate PD. Additional RCTs with large sample sizes are required to validate the efficacy of LSVT in patients with different progression of PD, including severe PD.

## Figures and Tables

**Table 1:** Characteristics of the studies included in the present meta-analysis.

Study (year)	Subject (N)	Age (mean (SD))	Sex M/F	HY	PD duration (years)	Intervention	Intervention/control	Outcomes
L O ramig 2001a	I = 21	I = 61.3 (11.4)	I = 17/4	I = 2.6 (0.6)	I = 7.2 (5.4)	I = LSVT	I = 1 h/time, 4 times/week, for 4 weeks	SPL, STSD
	C = 12	C = 63.3 (7.1)	C = 7/5	C = 2.2 (0.9)	C = 5.0 (4.6)	C = RET	C = 1 h/time, 4 times/week, for 4 weeks	

L O ramig 2001b	I = 14	I = 67.9 (9.0)	I = 7/07	—	I = 8.6 (6.3)	I = LSVT	I = 1 h/time, 4 times/week, for 4 weeks	SPL
	C = 14	C = 69.8 (6.8)	C = 7/07	—	—	C = NO	—	

LO ramig 1995	I = 26	I = 63.5 (11.5)	I = 21/05	I = 2.7 (0.7)	I = 8.3 (9.3)	I = LSVT	I = 4 times/week, for 4 weeks	SPL, STSD
	C = 19	C = 65.6 (8.9)	C = 12/07	C = 2.3 (0.8)	C = 5.9 (4.7)	C = RET	C = 4 times/week, for 4 weeks	Max duration

LO ramig 2018	I = 22	I = 64 (9)	I = 14/8	I = 2 (0.5)	I = 5 (4)	I = LSVT	I = 1 h/day, 4 days/week, for 4 weeks	SPL
	C = 20	C = 64 (9)	C = 13/7	—	—	C = NO	C = 45 min/day, once/week, for 6–8 weeks	

Arezzoo saffarian 2019	I = 13	I = 56.6 (4.70)	I = 7/6	I = 1.46 (0.49)	—	I = LSVT	I = 1 h/time, 4 days/week, for 4 weeks	VHI
	C = 13	C = 57.8 (3.46)	C = 5/5	C = 1.20 (0.34)	—	C = NO		

LO ramig 1996	I = 22	I = 63.23 (11.87)	—	I = 2.63 (0.6)	I = 6.55 (5.25)	I = LSVT	I = 16 times/month	SPL
	C = 13	C = 65.31 (8.89)	—	C = 2.25 (0.86)	C = 4.77 (3.06)	C = RET	C = 16 times/month	

Qi wu 2020	I = 50	I = 48.76 (7.21)	I = 22/28	—	I = 9.02 (3.71)	I = LSVT + Conventional	I = 30–40/times, 2 times/day, 5 weeks + 0–40 min/time, 3 times/day, 3 weeks	VHI, UPDRS-III, speech intelligibility
	C = 50	C = 48.76 (7.21)	C = 20/20	—	C = 9.68 (4.11)	C = Conventional	C = 30–40 min/time, 3 times/day, 3 weeks	

Haiyu tang 2016	I = 32	I = 69.75 (4.35)	I = 17/19	I = 2.02 (0.54)	I = 4.52 (3.26)	I = LSVT + Conventional	I = 40–60 min/time, 4 times/weeks, 12 weeks + 1 h/time, 4 times/week, 12 week	VHI, WAB, speech intelligibility
	C = 32	C = 68.45 (4.62)	C = 15/13	C = 2.05 (0.48)	C = 4.46 (3.43)	C = Conventional	C = 40–60 min/time, 4 times/week, 12 weeks	

Meifang yang 2017	I = 49	I = 66.83 (2.89)	I = 27/22	I = 2.11 (0.27)	I = 5.12 (0.67)	I = LSVT	I = 1 h/time, 4 times/week, 12 weeks	VHI, WAB
	C = 49	C = 65.82 (2.56)	C = 25/24	C = 2.09(0.34)	C = 5.52(0.16)	C = Conventional	C = 1 h/time, 4 times/week, 12 week	Speech intelligibility

Geralyn schulz 2021	I = 20	I = 67	I = 15/15	I = 2.13	I = 4.89	I = LSVT	—	SPL
	C = 18	C = 63.5	C = 11/07	C = 2.14	C = 4.64	C = NO	—	

I: intervention; C: control; LSVT: Lee Silverman voice treatment; RET: high-effort respiratory treatment program; NO: no treatment; SPL: sound press level; *S*TSD: semitone standard deviation; Max duration: maximum duration; PDQ-39 communication: Parkinson's disease questionnaire-39 communication; VHI: voice handicap index; WAB: western aphasia battery.

## Data Availability

The data used to support the findings of this study are available from the corresponding author upon request.
